# Supplementary material for: Quantum beats of exciton-polarons in CsPbI3 perovskite nanocrystals
Source: Nat Commun. 2026 May 26;17:4685. doi: 10.1038/s41467-026-73506-1 (PMC13212901; doi:10.1038/s41467-026-73506-1)
Supplement: Supplementary file 1 — Supplementary Information [file 41467_2026_73506_MOESM1_ESM.pdf]

Supplementary Information

Quantum beats of exciton-polarons in $CsPbI_3$ perovskite nanocrystals

Artur V. Trifonov, Mikhail O. Nestoklon, M. Alex Hollberg, Stefan Grisard, Dennis Kudlacik, Elena V. Kolobkova, Maria S. Kuznetsova, Serguei V. Goupalov, Jan M. Kaspari, Doris E. Reiter, Dmitri R. Yakovlev, Manfred Bayer, Ilya A. Akimov

Supplementary Note 1 Nanocrystals embedded in glass matrix

Most modern studies are focused on colloidal perovskite nanocrystals, which exhibit unique optical characteristics [1]. However, colloidal perovskite nanocrystals are relatively fragile which leads to degradation under exposure to environmental conditions, elevated temperatures, or high-power laser irradiation. This problem is partially resolved by various techniques of surface passivation, but the stability of the samples is not absolute. This limitation is mitigated in $CsPbX_3$ (X = Cl, Br, I) perovskite nanocrystals synthesized in a fluorophosphate glass matrix [2]. Such nanocrystals possess several advantages over their colloidal counterparts, including high stability of physical properties with respect to atmospheric exposure and heating, while preserving their characteristic optical and spin properties. The formation of $CsPbX_3$ perovskite nanocrystals in fluorophosphate glasses protects the nanocrystals from environmental influences and high-power laser irradiation, which results in the long-term stability of their optical properties. As an example, the sample studied in this work was synthesized in 2019 and has been investigated using multiple experimental techniques over the past six years, with no noticeable degradation observed. For the this sample the results were obtained using the following experimental techniques: Transmission [3, 4], photoluminescence [4–6], and transient spectrosciopy [6]; Time-resolved Faraday rotation [3, 4, 6, 7]; and Raman spectroscopy [5]. Thereby, these samples demonstrate excellent optical properties, including strong photoluminescence, transient pump–probe Faraday rotation, and Raman scattering signals, which have enabled studies of exciton and charge carrier spin dynamics in an external magnetic field [3, 4, 7], demonstration of hole spin mode locking [8], exciton interaction with confined acoustic phonons [5], and hyperfine interaction between charge carriers and nuclear spins [6] in lead halide perovkiste nanocrystals.

Nanocrystals embedded in the glass matrix retain their physical properties during repeated heating/cooling cycles up to 600 K. Regarding the quantum yield, nanocrystals embedded in fluorophosphate glasses are currently inferior to colloidal perovskite nanocrystals. In particular, $CsPbBr_3$ nanocrystals demonstrate a high and time-stable photoluminescence quantum yield exceeding 85% at room temperature [9]. For $CsPbI_3$ nanocrystals in a glass matrix similar to those studied in the present work, the quantum yield was previously about 5%; however, due to modification of the fluorophosphate matrix, it has now reached 25% (manuscript in preparation).

Note that the NC ensemble in these samples does not exhibit a Gaussian (normal) size distribution, which is commonly assumed for colloidal NCs. There is a number of

factors which might lead to multi-peak configuration of PL in Fig. 1a: (i) All luminescent phases of perovskite nanocrystals are metastable. Orthorhombic and cubic phases have a comparable probability of stabilization in the nanocrystalline state. The bandgap energies of these phases differ, which can result in two distinct nanocrystal ensembles contributing to the luminescence. (ii) The second emission band can be associated with the presence of shallow trap states which do not contribute to the coherent spectroscopy data. (iii) The cooling rate of the surface layers is significantly higher than that in the bulk of the sample, resulting in a non-uniform size distribution within the sample volume. However, we stress that the resonant excitation in PE technique allows us to study only an sub-ensemble of optically active NCs with distinct size. Different spectroscopic techniques do not always probe the same states as PL and can therefore show a significantly altered spectral response, especially for signals arising from exciton transitions in perovskites. For instance, in Raman spectroscopy the spectral range where Raman signal may be detected is much narrower than the full PL width, see Ref. [5], Figure 1a-d.

Supplementary Note 2 Evaluation of NC sizes

The fact that the nanocrystals are embedded in a glass matrix makes a reliable determination of their size and distribution by imaging techniques such as electron microscopy and X-ray diffraction highly challenging, see e.g. the scanning transmission electron microscopy images in Figs. S5 and S6 from Ref. [3]. Therefore, the size analysis for these samples was performed using optical spectroscopy methods based on the following approaches:

1. Spectral dependence of electron g-factors [3, 4]. It has been demonstrated that for electrons, a pronounced size-induced change of the g-factor occurs due to interaction of the bottom conduction band with the spin-orbit split electron states. This enables a correlation between the spectral dependence of the g-factor and the nanocrystal size.

2. Spectral dependence of Raman peak shifts for light scattering on confined acoustic phonons [5]. While optical phonon energies remain largely size-independent, the energies of confined acoustic phonons increase with decreasing NC size. By comparing experimental data with model predictions, it has been demonstrated that the nanocrystals predominantly have spherical or spheroidal shapes.

Based on that studies the photon energy can be recalculated into a NC diameter using the empirical expression $D = \sqrt{16.93/(E_\mathrm{X} - 1.652) - 4.31}$, where E_X is the position of exciton peak in eV.

Supplementary Table 1 Exciton lifetime and optical coherence time for perovskite nanocrystals reported in literature. In the table we also outline the method used to obtain the results and the material. PL - photoluminescence. PLE - photoluminescence excitation spectroscopy.

Source	method	system	T_1, ps	T_2, ps
Ref. [10]	photon correlation spectroscopy	$CsPbBr_3$	210	80
Ref. [11]	PL linewidth	$CsPbI_3$	—	116
Ref. [12]	time-resolved PL and PLE spectroscopy	$CsPbI_3$	1000	64
Ref. [6]	Time-resolved PL	$CsPbI_3$	500	—
Ref. [13]	photon echo	$CsPbBr_2Cl$	—	25
Ref. [14]	two-dimensional Fourier spectroscopy	$CsPbI_3$	—	5.5
This work	photon echo	$CsPbI_3$	600÷800	150÷330

Supplementary Note 3 Spectral dependence of optical coherence time T_2

Figure 1 shows the photon energy dependence of zero-phonon optical coherence time T_2 measured with picosecond (ps) and femtosecond (fs) laser pulses. The ps pulses with spectral full width at half maximum (FWHM) of about 0.5 meV provide a good spectral resolution of the overall smooth dependence of T_2 where it is gradually decreasing from 320 to 150 ps for increase of photon energy from 1.725 to 1.765 eV. For excitation with fs pulses (FWHM of 15 meV) similar values are obtained (see red diamonds in the Figure). This is in accordance with our expectations, because the spectral dependence is smooth and varies only weakly on the scale of the PE spectral width ($\approx$ 10 meV as estimated from PE transient profile). The data in Fig. 1 confirm that experiments with fs pulses are appropriate for evaluation of the size dependence of Huang-Rhys factors and phonon lifetimes, which are shown in Fig. 4. We note that the studied spectral range is relatively small, corresponding to a change in the quantum confinement energy from 73 meV to 113 meV only [3, 5]. Nevertheless, the latter corresponds to an approximately twofold change in the effective NC volume, leading to smooth but noticeable changes in T_2, $\tau_{\rm ph}$, and $S_{\rm HR}$.

The optical coherence time T_2 is smaller than the exciton lifetime limit of $2T_1$. The exciton lifetime in these NCs is given by $T_1 = 500$ ps as follows from time-resolved PL data in Ref. [6]. This indicates that the observed coherence time T_2 is not limited by lifetime broadening but is instead governed by phase relaxation processes (pure decoherence). The strong temperature sensitivity, i.e. the decrease of T_2 from $\sim$ 300 ps to $\sim$ 50 ps upon heating from 2 K to 5 K, clearly points to phonon-related mechanisms. It is therefore reasonable to assume that the observed spectral dependence of T_2 is determined by the specifics of exciton–phonon coupling, most likely involving acoustic phonons with energies of 0.5–1 meV at such low temperatures. One possible scenario is that changes in the nanocrystal size modify the degree of resonant interaction between acoustic phonons and the fine structure split excitons, consistent with Ref. [15].

In Table 1 we review recent reports on measured exciton lifetieme T_1 and exciton optical coherence time T_2. Note that the measurements in Refs. [10–12] were performed on single colloidal nanocrystals.

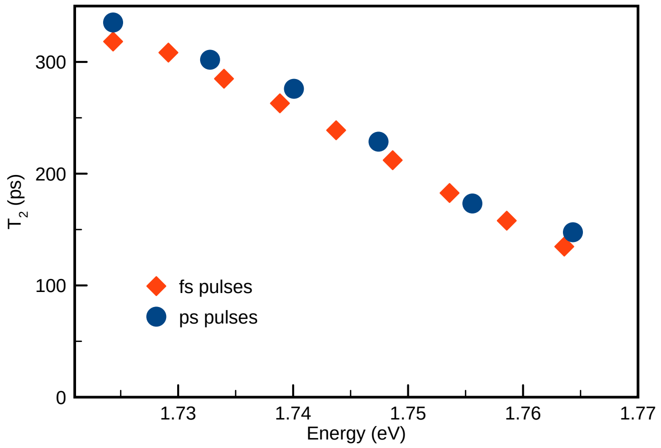

Supplementary Fig. 1 Spectral dependence of zero-phonon optical coherence time T_2 under excitation with spectrally narrow (circles) picosecond and broad fs pulses (diamonds).

Supplementary Note 4 Fine structure of exciton

To take into account the fine structure of the exciton, we assume that the three-fold bright exciton state is split into mutually orthogonal linearly polarized components. For the photon echo calculations the following procedure is used: the dynamics is calculated in the eigenbasis of NC $\{|0\rangle, |x'\rangle, |y'\rangle, |z'\rangle\}$ then the equations for the density matrix components are rotated to the laboratory frame $\{|0\rangle, |x\rangle, |y\rangle, |z\rangle\}$. The 1st pulse is assumed to be polarized along z axis, the 2nd pulse at time τ_{12} is either polarized along z or x axis for HHH and HVH schemes respectively. The polarization along z is detected at the photon echo time $2\tau_{12}$. The orientation of a nanocrystal in the laboratory reference frame is described by three Euler angles α, β, and γ. The rotation matrix relating coordinates in xyz and $x'y'z'$ bases is [16]:

$$R(\alpha,\beta,\gamma) = \begin{pmatrix} c_\alpha c_\beta c_\gamma - s_\alpha s_\gamma & -c_\alpha c_\beta s_\gamma - s_\alpha c_\gamma & c_\alpha s_\beta \\ s_\alpha c_\beta c_\gamma + c_\alpha s_\gamma & -s_\alpha c_\beta s_\gamma + c_\alpha c_\gamma & s_\alpha s_\beta \\ -s_\beta c_\gamma & s_\beta s_\gamma & c_\beta \end{pmatrix}, \tag{1}$$

here and below in this section we use $s_\xi \equiv \sin\xi$, $c_\xi \equiv \cos\xi$ ($\xi = \alpha,\beta,\gamma$) to shorten the notation.

In the eigenbasis of NC, the dynamics is given by:

$$\rho_{0x'} = e^{-t/T_2} e^{i\omega_1 t} \rho^0_{0x'}\,, \qquad \rho_{0y'} = e^{-t/T_2} e^{i\omega_2 t} \rho^0_{0y'}\,, \qquad \rho_{0z'} = e^{-t/T_2} e^{i\omega_3 t} \rho^0_{0z'}\,; \tag{2}$$

$$\rho_{x'0} = e^{-t/T_2} e^{-i\omega_1 t} \rho^0_{x'0}\,, \qquad \rho_{y'0} = e^{-t/T_2} e^{-i\omega_2 t} \rho^0_{y'0}\,, \qquad \rho_{z'0} = e^{-t/T_2} e^{-i\omega_3 t} \rho^0_{z'0}\,. \tag{3}$$

Here $\hbar\omega_i$ ($i = 1, 2, 3$) are the three exciton energies for excitons linerly polarized along x', y', z' respectively. We assume that the diplole matrix element (and, consequently, radiative decay time) as well as the decoherence time T_2 is the same for all three

exciton states. After 1st pulse the components of density matrix are

$$\rho_{0z}^{1+} = 1\,, \quad \rho_{0y}^{1+} = \rho_{0x}^{1+} = 0\,. \tag{4}$$

After 2nd pulse we have two options: for HHH scheme

$$\rho_{z0}^{2+} = \rho_{0z}^{2-}\,, \quad \rho_{y0}^{2+} = \rho_{x0}^{2+} = 0\,. \tag{5}$$

and for HVH scheme

$$\rho_{x0}^{2+} = \rho_{0x}^{2-}\,, \quad \rho_{y0}^{2+} = \rho_{z0}^{2+} = 0\,. \tag{6}$$

Rotating the dynamics equations (2) into laboratory coordinate frame using (1) gives rather lengthy equations for the amplitude of the signal:

$$A_{\parallel}(\alpha,\beta,\gamma) \propto \rho_{z0} = \big[c_\alpha^4 s_\beta^4 + s_\alpha^4 s_\beta^4 + c_\beta^4 \\ +2s_\alpha^2 c_\alpha^2 s_\beta^4 \cos(\omega_1-\omega_2)\tau_{12} + 2c_\alpha^2 s_\beta^2 c_\beta^2 \cos(\omega_1-\omega_3)\tau_{12} + 2s_\alpha^2 s_\beta^2 c_\beta^2 \cos(\omega_2-\omega_3)\tau_{12}\big] e^{-2\tau_{12}/T_2} \tag{7}$$

$$A_\times(\alpha,\beta,\gamma) \propto \rho_{z0} = \big[c_\alpha^2 s_\beta^2 (c_\alpha c_\beta c_\gamma - s_\alpha s_\gamma)^2 + s_\alpha^2 s_\beta^2 (s_\alpha c_\beta c_\gamma + c_\alpha s_\gamma)^2 + s_\beta^2 c_\beta^2 c_\gamma^2 \\ +2s_\alpha c_\alpha s_\beta^2 (c_\alpha c_\beta c_\gamma - s_\alpha s_\gamma)(s_\alpha c_\beta c_\gamma + c_\alpha s_\gamma)\cos(\omega_1-\omega_2)\tau_{12} - 2c_\alpha s_\beta^2 c_\beta c_\gamma (c_\alpha c_\beta c_\gamma - s_\alpha s_\gamma)\cos(\omega_1-\omega_3)\tau_{12} \\ - 2s_\alpha s_\beta^2 c_\beta c_\gamma (s_\alpha c_\beta c_\gamma + s_\alpha s_\gamma)\cos(\omega_2-\omega_3)\tau_{12}\big] e^{-2\tau_{12}/T_2} \tag{8}$$

The result should be averaged over directions using:

$$\langle A \rangle_{\text{ang}} \equiv \frac{1}{8\pi^2} \int_0^{2\pi} \mathrm{d}\alpha \int_0^{\pi} \sin\beta \mathrm{d}\beta \int_0^{2\pi} \mathrm{d}\gamma\; A(\alpha,\beta,\gamma) \tag{9}$$

Assuming the constant energies of three exciton components, but the arbitrary direction of the NCs, the amplitude of the echo is proportional to

$$\langle A_\parallel \rangle_{\text{ang}} \propto \left[\frac{6}{5} + \frac{4}{15}\sum_{i>j}\cos(\omega_i-\omega_j)\tau_{12}\right] e^{-\frac{2\tau_{12}}{T_2}}\,, \tag{10}$$

$$\langle A_\times \rangle_{\text{ang}} \propto \left[\frac{2}{5} - \frac{2}{15}\sum_{i>j}\cos(\omega_i-\omega_j)\tau_{12}\right] e^{-\frac{2\tau_{12}}{T_2}}\,. \tag{11}$$

Now we also take into account the possible deviation of exciton energies. The absolute value of the exciton energy is not important for the phonon echo, only the splittings. E.g., the amplitude averaged over distribution of splittings between 1st and 2nd levels $\omega_{12} = \omega_1 - \omega_2$ is given by

$$\langle A \rangle_{\omega_{12}} = \int W(\omega_{12}) A(\omega_{12}, \omega_{13}, \omega_{23} = \omega_{12} - \omega_{13}) d\omega_{12} \tag{12}$$

Assuming the distribution function of the splittings to be Lorentzian,

$$W(\omega) = \frac{t_i^*}{2\pi} \frac{1}{(\omega - \delta_i)^2 + 4/(t_i^*)^2}, \tag{13}$$

and that all three splittings are not correlated, the PE signal averaged over both the angles and the splittings distribution is given by

$$\langle A_\parallel \rangle \propto \left[\frac{18}{15} + \frac{4}{15} \sum_{i=1,2,3} \cos(\delta_i \tau_{12}) e^{-\frac{2\tau_{12}}{t_i^*}} \right] e^{-\frac{2\tau_{12}}{T_2}}, \tag{14}$$

$$\langle A_\times \rangle \propto \left[\frac{6}{15} - \frac{2}{15} \sum_{i=1,2,3} \cos(\delta_i \tau_{12}) e^{-\frac{2\tau_{12}}{t_i^*}} \right] e^{-\frac{2\tau_{12}}{T_2}}, \tag{15}$$

where the parameters t_i^* work as an effective signal dephasing times and δ_i are the average splittings. An interesting result is that even though the PE signal is stable with respect to the level disorder, the disorder of its fine structure gives the contribution to the additional decay, not of the full signal, but of its oscillating components.

Supplementary Note 5 Model of photon echo of exciton-polaron states

In this section we outline the derivation of PE signal in the exciton-polaron model. We assume the exciton-polaron with relatively small Huang-Rhys factor which allows to perform calculations in four (six with account on light polarization) level scheme.

Scheme of the levels and dynamics of the system

We consider the four-level system shown in Fig. 3b. There are: the state with zero excitons and phonons $|0\rangle$ (crystal ground state), the state without exciton but with one phonon $|0'\rangle$, the ground state of exciton-polaron, with no phonons $|X\rangle$, and the excited exciton-polaron state with one phonon $|X'\rangle$. The energies of the corresponding states are 0, $\hbar\Omega$, $\hbar\omega_X$, $\hbar\omega_X + \hbar\Omega$. Note that the excitons and phonons discussed here are localised in the NC, and the details of free polarons dynamics relevant for bulk perovskites [17] or 2D perovskite semiconductors [18] may be neglected. In addition we neglect interactions between NCs, either through photons [19] or phonons [20]. In the calculations below we assume that the spectral width of the laser pulse covers at least all optical transitions between the levels described above.

For exciton-polarons (in contrast to non-interacting excitons), the optically allowed transitions are not only $0 \leftrightarrow X$ (with probability γ_0) and $0' \leftrightarrow X'$ (with probability γ_1), but also $0' \leftrightarrow X$ and $0 \leftrightarrow X'$ (in this model, probability γ' is the same for both transitions). In exciton-polaron model [21, 22], the transition probability is proportional to product of dipole matrix element $|\mathbf{d}|^2$ and the overlap of oscillator functions shifted due to exciton formation which leads to extra factor

$$\gamma_0 \propto |\mathbf{d}|^2 e^{-2S_{HR}} \tag{16a}$$

$$\gamma' \propto |\mathbf{d}|^2 S_{HR} e^{-2S_{HR}} \tag{16b}$$
$$\gamma_1 \propto |\mathbf{d}|^2 (1 - S_{HR})^2 e^{-2S_{HR}} \tag{16c}$$

where $\mathbf{d}$ is the dipole matrix elements of the optical transition, and we defined Huang-Rhys factor as $S_{HR} = \epsilon_{pol}/(\hbar\Omega)$ (ϵ_{pol} is the polaron formation energy). Note that in this approximation polaron formation energy should be small which means that $\gamma' \ll \gamma_0, \gamma_1$.

In addition, we assume that the phonon may decay, which leads to transitions $0' \to 0$ and $X' \to X$ with the same probability γ_{ph}.

To trace the polarization of the signal, we need to consider two polarization states of exciton, from now on the enumeration 0 and $0'$ are ground state and state with one phonon, 1(2) and $1'(2')$ are right(left) circularly polarized states of ground state exciton-polaron and the excited state of exciton-polaron, respectively. This makes the model effectively six-level. Still, we call the model four-level as here we neglect the splittings between levels (as the role of the fine structure is excluded, see Sec. 4) and we need it only to demonstrate that the phonon structure does not change the polarization structure of the signal. Below, we extensively omit states 2 and $2'$ when equations are same.

The Lindblad equation, with all levels considered is:

$$\frac{\mathrm{d}\rho}{\mathrm{d}t} = -\frac{i}{\hbar}[H_0, \rho] + \mathcal{L}_d[\hat{\rho}] + \Gamma \odot \hat{\rho}\,. \tag{17}$$

For convenience and compactness of the notation, with the use of $\odot$ for the Hadamard product (element-wise product or Schur product).

The Hamiltonian is

$$H_0 = \begin{pmatrix} 0 & 0 & 0 & 0 & 0 & 0 \\ 0 & \hbar\Omega & 0 & 0 & 0 & 0 \\ 0 & 0 & \hbar\omega_{\mathrm{X}} & 0 & 0 & 0 \\ 0 & 0 & 0 & \hbar\omega_{\mathrm{X}} & 0 & 0 \\ 0 & 0 & 0 & 0 & \hbar\omega_{\mathrm{X}} + \hbar\Omega & 0 \\ 0 & 0 & 0 & 0 & 0 & \hbar\omega_{\mathrm{X}} + \hbar\Omega \end{pmatrix} \tag{18}$$

and the “inflow”diagonal part of superoperator is

$$\mathcal{L}_d[\hat{\rho}] = \begin{pmatrix} \gamma_{\mathrm{ph}}\rho_{0'0'} + \gamma_0\rho_X + \gamma'\rho_{X'} & 0 & 0 & 0 & 0 & 0 \\ 0 & \gamma_1\rho_{X'} + \gamma'\rho_X & 0 & 0 & 0 & 0 \\ 0 & 0 & \gamma_{\mathrm{ph}}\rho_{1'1'} & 0 & 0 & 0 \\ 0 & 0 & 0 & \gamma_{\mathrm{ph}}\rho_{2'2'} & 0 & 0 \\ 0 & 0 & 0 & 0 & 0 & 0 \\ 0 & 0 & 0 & 0 & 0 & 0 \end{pmatrix} \tag{19}$$

where for convenience we introduced $\rho_X = \rho_{11} + \rho_{22}$ and $\rho_{X'} = \rho_{1'1'} + \rho_{2'2'}$, and the matrix in the "outflow" part is

$$\Gamma = -\frac{1}{2}\begin{pmatrix} 0 & \gamma_{\text{ph}} & \gamma_0' & \gamma_0' & \gamma_{\text{ph}}+\gamma_1' & \gamma_{\text{ph}}+\gamma_1' \\ \gamma_{\text{ph}} & 2\gamma_{\text{ph}} & \gamma_{\text{ph}}+\gamma_0' & \gamma_{\text{ph}}+\gamma_0' & 2\gamma_{\text{ph}}+\gamma_1' & 2\gamma_{\text{ph}}+\gamma_1' \\ \gamma_0' & \gamma_{\text{ph}}+\gamma_0' & 2\gamma_0' & 2\gamma_0' & \gamma_{\text{ph}}+\gamma_1'+\gamma_0' & \gamma_{\text{ph}}+\gamma_0'+\gamma_1' \\ \gamma_0' & \gamma_{\text{ph}}+\gamma_0' & 2\gamma_0' & 2\gamma_0' & \gamma_{\text{ph}}+\gamma_1'+\gamma_0' & \gamma_{\text{ph}}+\gamma_0'+\gamma_1' \\ \gamma_{\text{ph}}+\gamma_1' & 2\gamma_{\text{ph}}+\gamma_1' & \gamma_{\text{ph}}+\gamma_0'+\gamma_1' & \gamma_{\text{ph}}+\gamma_0'+\gamma_1' & 2\gamma_{\text{ph}}+2\gamma_1' & 2\gamma_{\text{ph}}+2\gamma_1' \\ \gamma_{\text{ph}}+\gamma_1' & 2\gamma_{\text{ph}}+\gamma_1' & \gamma_{\text{ph}}+\gamma_0'+\gamma_1' & \gamma_{\text{ph}}+\gamma_0'+\gamma_1' & 2\gamma_{\text{ph}}+2\gamma_1' & 2\gamma_{\text{ph}}+2\gamma_1' \end{pmatrix} \tag{20}$$

where for convenience we defined

$$\gamma_0' = \gamma_0 + \gamma' \,, \quad \gamma_1' = \gamma_1 + \gamma' \,. \tag{21}$$

Effect of the pulse on density matrix

Now we consider light-mediated transitions in the system shown in Fig. 3b. This is again generalization of the two-level system, check e.g. chapters 2, 6 and 10 of [23]. The pulse is approximated by the plane wave with the smooth enveope $\mathbf{E}_0(z,t)$ and electric field given by

$$\mathbf{E}(\mathbf{r},t) = \mathbf{E}_0(\mathbf{k}\cdot\mathbf{r}/k, t)e^{i(\mathbf{k}\cdot\mathbf{r}-\omega t)} + \text{c.c.} \tag{22}$$

Assuming the pulse duration is short compared with all coherence and relaxation times, the evolution of density matrix (DM) during the pulse may be found from the von-Neumann equation

$$\frac{\mathrm{d}\rho}{\mathrm{d}t} = -\frac{\mathrm{i}}{\hbar}\left[H_0 + V, \rho\right] \,, \tag{23}$$

where, in rotating wave approximation,

$$V = \hbar\begin{pmatrix} 0 & 0 & \tilde{f}_+^* & \tilde{f}_-^* & s\cdot\tilde{f}_+^* & s\cdot\tilde{f}_-^* \\ 0 & 0 & s\cdot\tilde{f}_+^* & s\cdot\tilde{f}_-^* & \tilde{f}_+^* & \tilde{f}_-^* \\ \tilde{f}_+ & s\cdot\tilde{f}_+ & 0 & 0 & 0 & 0 \\ \tilde{f}_- & s\cdot\tilde{f}_- & 0 & 0 & 0 & 0 \\ s\cdot\tilde{f}_+ & \tilde{f}_+ & 0 & 0 & 0 & 0 \\ s\cdot\tilde{f}_- & \tilde{f}_- & 0 & 0 & 0 & 0 \end{pmatrix} \,, \tag{24}$$

$$\tilde{f}_\pm = dE_0^\pm e^{-i\omega t} \equiv f_\pm e^{-i\omega t} \,, \tag{25}$$

where d is the dipole matrix element for the transitions $0 \leftrightarrow 1,2$, $s = \sqrt{S_{HR}}$, $E_0^\pm$ are circular components of the envelope (22).

Solution of Eq. (23) is surprisingly complex in general case, for two-level system it may be found in Ref. [23], but we are interested in four-level system and the case of small pulse area when the full solution is surplus. When the pulse area $\theta_\pm$ (equal to $f_\pm t_p$ for square pulse of duration t_p) is small compared with Rabi frequency, the first

order correction to the density matrix may be obtained as

$$\rho^{+} = -\frac{i}{\hbar}\left[H_0 + V, \rho^{-}\right] t_p \equiv \hat{\mathcal{A}}\rho^{-} \,, \tag{26}$$

where ρ^{-} is the density matrix before pulse and ρ^{+} is the density matrix after pulse. Full solution of Eq. (26) is too lengthy for our needs, below we will only give the results relevant for the calculations.

For the PE we will need the action of the light pulse in the second order over pulse area. It may be shown that up to second order, it may be calculated as

$$\rho^{+} = \hat{\mathcal{A}}\hat{\mathcal{A}}\rho^{-} \,. \tag{27}$$

Below we also give only part of this result relevant for photon echo calculations.

Photon echo

The signal in two circular polarizations is proportional [23] to the corresponding components of polarization vector which is given by the following components of the density matrix:

$$P_{+} \sim \rho_{10} + \rho_{1'0'} + s\rho_{1'0} + s\rho_{10'} \,, \tag{28a}$$

$$P_{-} \sim \rho_{20} + \rho_{2'0'} + s\rho_{2'0} + s\rho_{20'} \,. \tag{28b}$$

Note the extra factor s in Eq. (28) which originates from the ratio of diplole matrix elements for phonon-assisted transitions to the phonon-less transitions. Thus, we are interested in the following components of the density matrix at times after second pulse: ρ_{10}, ρ_{20}, $\rho_{1'0}$, $\rho_{2'0}$, $\rho_{10'}$, $\rho_{20'}$, $\rho_{1'0'}$, $\rho_{2'0'}$.

Density matrix after 1st pulse

The components of DM after 1st pulse are given by (26) which leads to:

$$\rho_{01}^{1+} = i\theta_{1+}^{*} \,, \quad \rho_{01'}^{1+} = is\theta_{1+}^{*} \,, \quad \rho_{02}^{1+} = i\theta_{1-}^{*} \,, \quad \rho_{02'}^{1+} = is\theta_{1-}^{*} \,. \tag{29}$$

Relaxation of DM components after 1st impulse

One needs to trace the evolution of the following DM components: ρ_{01}, $\rho_{01'}$, ρ_{02}, $\rho_{02'}$. Below we give results only for right polarization, dynamics of other components is the same. Writing the components of the Lindblad equation (17), we obtain the following dynamics of the DM components:

$$\rho_{01}\left(t \in (0, \tau_{12})\right) = \rho_{01}^{1+} e^{i\omega_{\mathrm{X}} t} e^{-\frac{\gamma_0'}{2} t} \,, \tag{30a}$$

$$\rho_{01'}\left(t \in (0, \tau_{12})\right) = \rho_{01'}^{1+} e^{i(\omega_{\mathrm{X}} + \Omega) t} e^{-\frac{\gamma_1' + \gamma_{\mathrm{ph}}}{2} t} \,, \tag{30b}$$

This leads to the following components of the DM at the time of 2nd pulse $t = \tau_{12}$

$$\rho_{01}^{2-} = \rho_{01}^{1+} \varepsilon_{\mathrm{X}} \Gamma_0 \,, \quad \rho_{01'}^{2-} = \rho_{01'}^{1+} \varepsilon_{\mathrm{X}} \varepsilon \Gamma_1 \Gamma_p \,, \tag{31}$$

where for convenience we defined

$$\varepsilon_\mathrm{X} = e^{i\omega_\mathrm{X}\tau_{12}}\,, \quad \varepsilon = e^{i\Omega\tau_{12}}\,, \quad \Gamma_0 = e^{-\frac{\gamma_0'}{2}\tau_{12}}\,, \quad \Gamma_1 = e^{-\frac{\gamma_1'}{2}\tau_{12}}\,, \quad \Gamma_p = e^{-\frac{\gamma_\mathrm{ph}}{2}\tau_{12}}\,. \tag{32}$$

Note that Γ_0 and Γ_1 are almost equal and defined by the exciton coherence time (under our assumptions half of its lifetime). The difference between them is $\Gamma_1/\Gamma_0 = e^{-\frac{\gamma_1-\gamma_0}{2}\tau_{12}} = e^{\frac{S_{HR}\gamma_0}{2}\tau_{12}}$ which is much smaller than all typical times in the system since S_{HR} is small.

DM after 2nd pulse

From (27) we may compute the components relevant for the PE signal after the 2nd pulse:

$$\rho_{10}^{2+} = \left[\theta_{2+}^2(\rho_{01}^{2-} + s\rho_{01'}^{2-}) + \theta_{2+}\theta_{2-}(\rho_{02}^{2-} + s\rho_{02'}^{2-})\right] \tag{33a}$$
$$\rho_{10'}^{2+} = \left[\theta_{2+}^2(s\rho_{01}^{2-} + \rho_{01'}^{2-}) + \theta_{2+}\theta_{2-}(s\rho_{02}^{2-} + \rho_{02'}^{2-})\right] \tag{33b}$$
$$\rho_{1'0}^{2+} = s\left[\theta_{2+}^2(\rho_{01}^{2-} + s\rho_{01'}^{2-}) + \theta_{2+}\theta_{2-}(\rho_{02}^{2-} + s\rho_{02'}^{2-})\right] \tag{33c}$$
$$\rho_{1'0'}^{2+} = s\left[\theta_{2+}^2(\rho_{01'}^{2-} + s\rho_{01}^{2-}) + \theta_{2+}\theta_{2-}(\rho_{02'}^{2-} + s\rho_{02}^{2-})\right] \tag{33d}$$

and

$$\rho_{20}^{2+} = \left[\theta_{2+}\theta_{2-}(\rho_{01}^{2-} + s\rho_{01'}^{2-}) + \theta_{2-}^2(\rho_{02}^{2-} + s\rho_{02'}^{2-})\right] \tag{34a}$$
$$\rho_{2'0}^{2+} = \left[\theta_{2+}\theta_{2-}(s\rho_{01}^{2-} + \rho_{01'}^{2-}) + \theta_{2-}^2(s\rho_{02}^{2-} + \rho_{02'}^{2-})\right] \tag{34b}$$
$$\rho_{20'}^{2+} = s\left[\theta_{2+}\theta_{2-}(\rho_{01}^{2-} + s\rho_{01'}^{2-}) + \theta_{2-}^2(\rho_{02}^{2-} + s\rho_{02'}^{2-})\right] \tag{34c}$$
$$\rho_{2'0'}^{2+} = s\left[\theta_{2+}\theta_{2-}(\rho_{01'}^{2-} + s\rho_{01}^{2-}) + \theta_{2-}^2(\rho_{02'}^{2-} + s\rho_{02}^{2-})\right] \tag{34d}$$

Relaxation of DM components after 2nd impulse

We we need to know the evolution of the DM components which enter (28). Below we give only components entering P_+. Dynamics of components entering P_- is the same.

Writing the components of the Lindblad equation (17), we obtain the following dynamics of the DM components:

$$\rho_{10}\,(t>t_2) = \rho_{10}^{2+}e^{-i\omega_\mathrm{X}(t-t_2)}e^{-\frac{\gamma_0'}{2}(t-t_2)}\,, \tag{35a}$$
$$\rho_{1'0}\,(t>t_2) = \rho_{1'0}^{2+}e^{-i(\omega_\mathrm{X}+\Omega)(t-t_2)}e^{-\frac{\gamma_1'+\gamma_\mathrm{ph}}{2}(t-t_2)}\,, \tag{35b}$$
$$\rho_{10'}\,(t>t_2) = \rho_{10'}^{2+}e^{-i(\omega_\mathrm{X}-\Omega)(t-t_2)}e^{-\frac{\gamma_0'+\gamma_\mathrm{ph}}{2}(t-t_2)}\,, \tag{35c}$$
$$\rho_{1'0'}\,(t>t_2) = \rho_{1'0'}^{2+}e^{-i\omega_\mathrm{X}(t-t_2)}e^{-\frac{\gamma_1'+2\gamma_\mathrm{ph}}{2}(t-t_2)}\,, \tag{35d}$$

Which gives the DM components at the time of echo signal $t - t_2 = \tau_{12}$:

$$\rho_{10}^{PE} = \rho_{10}^{2+}\varepsilon_\mathrm{X}^*\Gamma_0\,, \qquad \rho_{1'0}^{PE} = \rho_{1'0}^{2+}\varepsilon_\mathrm{X}^*\varepsilon^*\Gamma_1\Gamma_p\,, \tag{36a}$$
$$\rho_{10'}^{PE} = \rho_{10'}^{2+}\varepsilon_\mathrm{X}^*\varepsilon\Gamma_0\Gamma_p\,, \qquad \rho_{1'0'}^{PE} = \rho_{1'0'}^{2+}\varepsilon_\mathrm{X}^*\Gamma_1\Gamma_p^2\,. \tag{36b}$$

The evolution of components corresponding to second circular polarization is the same.

Contribution of different paths to PE

To get the final result, we substitude (29) into (31) and then the result into (33) and then from (36) we get the DM at the time of PE. The amplitude of the signal in two polarizations is then obtained from Eqs. (28):

$$P_+ \sim \rho_{10}^{\mathrm{PE}} + \rho_{1'0'}^{\mathrm{PE}} + s\rho_{1'0}^{\mathrm{PE}} + s\rho_{10'}^{\mathrm{PE}} \,, \tag{37a}$$

$$P_- \sim \rho_{20}^{\mathrm{PE}} + \rho_{2'0'}^{\mathrm{PE}} + s\rho_{2'0}^{\mathrm{PE}} + s\rho_{20'}^{\mathrm{PE}} \,. \tag{37b}$$

Collecting all terms in the result we have for contributions to P_+ from ρ_{10}, $\rho_{1'0'}$, $\rho_{10'}$, $\rho_{1'0}$ originating from the DM components after 1st pulse given below in the beginning of each line:

$$\rho_{01,02} \to \rho_{10}^{\mathrm{PE}} \qquad \Gamma_0^2 A_+ \,, \tag{38a}$$

$$\rho_{01',02'} \to \rho_{10}^{\mathrm{PE}} \qquad s^2\varepsilon\Gamma_0\Gamma_1\Gamma_p A_+ \,, \tag{38b}$$

$$\rho_{01,02} \to \rho_{10'}^{\mathrm{PE}} \qquad s^2\varepsilon\Gamma_0^2\Gamma_p A_+ \,, \tag{38c}$$

$$\rho_{01',02'} \to \rho_{10'}^{\mathrm{PE}} \qquad s^2\varepsilon^2\Gamma_0\Gamma_1\Gamma_p^2 A_+ \,, \tag{38d}$$

$$\rho_{01,02} \to \rho_{1'0}^{\mathrm{PE}} \qquad s^2\varepsilon^*\Gamma_0\Gamma_1\Gamma_p A_+ \,, \tag{38e}$$

$$\rho_{01',02'} \to \rho_{1'0}^{\mathrm{PE}} \qquad s^4\Gamma_1^2\Gamma_p^2 A_+ \,, \tag{38f}$$

$$\rho_{01,02} \to \rho_{1'0'}^{\mathrm{PE}} \qquad s^2\Gamma_0\Gamma_1\Gamma_p^2 A_+ \,, \tag{38g}$$

$$\rho_{01',02'} \to \rho_{1'0'}^{\mathrm{PE}} \qquad s^2\varepsilon\Gamma_1^2\Gamma_p^3 A_+ \,, \tag{38h}$$

where

$$A_+ = \theta_{2+}\left(\theta_{2+}\theta_{1+}^* + \theta_{2-}\theta_{1-}^*\right) \,. \tag{39a}$$

Note that the term (38f) may be dropped as it exceeds precision of equations.

The contribution to P_- from ρ_{20}, $\rho_{2'0'}$, $\rho_{20'}$, $\rho_{2'0}$ is the same as (38) but instead of A_+ the polarization dependence is given by

$$A_- = \theta_{2-}\left(\theta_{2-}\theta_{1-}^* + \theta_{2+}\theta_{1+}^*\right) \,. \tag{39b}$$

It is convenient to write both as

$$A_\pm = \theta_{2\pm}A_0 \,, \quad A_0 = \theta_{2-}\theta_{1-}^* + \theta_{2+}\theta_{1+}^* \,, \tag{40}$$

to see that the polarization dependence of the PE signal coincides with second impulse polarization while its amplitude depends on respective polarization of first and second pulses.

As a result, the amplitude of the non-oscillating signal is

$$\mathcal{P}_\pm^{PE,0} \sim \left[\Gamma_0^2 + S_{HR}\Gamma_0\Gamma_1\Gamma_p^2\right] A_\pm \,. \tag{41}$$

The amplitude of the signal oscillating at Ω is

$$\mathcal{P}_{\pm}^{PE,\Omega} \sim S_{HR}\Gamma_p \left[\Gamma_0\Gamma_1 \left(\varepsilon + \varepsilon^*\right) + \varepsilon \left(\Gamma_0^2 + \Gamma_1^2\Gamma_p^2\right)\right] A_{\pm} \,. \tag{42}$$

and of the signal oscillating at 2Ω is

$$\mathcal{P}_{\pm}^{PE,2\Omega} \sim S_{HR}\Gamma_0\Gamma_1\Gamma_p^2\varepsilon^2 A_{\pm} \,. \tag{43}$$

Result

Let us first consider the general scheme of the polarization dependence of PE signal. Following [24], we consider linearly polarized excitation pulses with second pulse rotated to angle ϕ and linearly polarized detection with polarized rotated to ϕ_d. Then

$$\theta_{1+} = \theta_{1-} = 1/\sqrt{2}\,, \quad \theta_{2+} = e^{i\phi}/\sqrt{2}\,, \quad \theta_{2-} = e^{-i\phi}/\sqrt{2}\,. \tag{44}$$

Which gives $A_0 = \cos\phi$ and

$$A_{\pm} = \frac{e^{\pm i\phi}}{\sqrt{2}} \cos\phi \,. \tag{45}$$

Amplitude of linearly-polarized heterodyne detection of main PE signal is proportional to

$$P_d^0 = \left| \frac{e^{i(\phi-\phi_d)} + e^{-i(\phi_d-\phi)}}{2} \cos\phi \right| = \left|\cos\left(\phi - \phi_d\right)\cos\phi\right| \tag{46}$$

We have two configurations: HHH and HVH. First is $\phi = \phi_d = 0$ and second is $\phi_d = 0$, $\phi = \pi/2$. This gives

$$P_d^{0,HHH} = 1\,, \quad P_d^{0,HVH} = 0. \tag{47}$$

As long as polarization dependence on the excitation/detection angles (46) is real (which is the case for HHH and HVH configurations), the absolute value of product is the product of absolute values and signal is zero for HVH scheme and for HHH scheme (we omit index HHH below) is:

$$\mathcal{P}_d^{PE} = \Gamma_0^2 \left| \left[1 + S_{HR}\widetilde{\Gamma}\Gamma_p^2\right] + S_{HR}\Gamma_p \left[\widetilde{\Gamma}\left(\varepsilon + \varepsilon^*\right) + \varepsilon(1 + \widetilde{\Gamma}^2\Gamma_p^2)\right] + S_{HR}\widetilde{\Gamma}\Gamma_p^2\varepsilon^2 \right| \tag{48}$$

where we introduced

$$\widetilde{\Gamma} = \Gamma_1/\Gamma_0 = e^{S_{HR}\gamma_0\tau_{12}} \,. \tag{49}$$

Note that $\widetilde{\Gamma}$ is 1 as compared with both, Γ_0 and Γ_p.

To write explicitly the result for the PE amplitude, we use the fact that for $t \ll 1$ and arbitrary complex ϵ:

$$|1 + t\epsilon| = 1 + t\mathrm{Re}(\epsilon) + \frac{t^2}{2}\left(\mathrm{Im}(\epsilon)\right)^2 + O\left(t^3\right) \,. \tag{50}$$

We keep second order terms to highlight the fact that even for single frequency of the complex amplitude, the absolute value in (48) will result in second order in S_{HR} signal at double frequency.

Using (50) we may write the final result (keeping only terms to the first order in S_{HR})

$$\mathcal{P}_d^{PE} = \Gamma_0^2 \left\{ 1 + S_{HR}\Gamma_p \left[\widetilde{\Gamma}\Gamma_p + \left(1 + 2\widetilde{\Gamma} + \widetilde{\Gamma}^2\Gamma_p^2\right)\cos(\Omega\tau_{12}) + \widetilde{\Gamma}\Gamma_p \cos(2\Omega\tau_{12}) \right] \right\} \tag{51}$$

Or, explicitly,

$$\begin{aligned}\mathcal{P}_d^{PE} = e^{-\gamma_0'\tau_{12}} \Bigg[1 + S_{HR} e^{-\frac{\gamma_{\rm ph}}{2}\tau_{12}} \Bigg(& e^{-\frac{\gamma_{\rm ph}-2S_{HR}\gamma_0}{2}\tau_{12}} \\ & + \left(1 + 2e^{S_{HR}\gamma_0\tau_{12}} + e^{-(\gamma_{\rm ph}-2S_{HR}\gamma_0)\tau_{12}}\right)\cos(\Omega\tau_{12}) \\ & + e^{-\frac{\gamma_{\rm ph}-2S_{HR}\gamma_0}{2}\tau_{12}} \cos(2\Omega\tau_{12}) \Bigg) \Bigg] ,\end{aligned} \tag{52}$$

and, finally, with the help of Eqs.(16,21):

$$\begin{aligned}\mathcal{P}_d^{PE} = e^{-(1+S_{HR})\gamma_0\tau_{12}} \Bigg[& 1 + S_{HR} e^{-(\gamma_{\rm ph}-S_{HR}\gamma_0)\tau_{12}} \\ & + S_{HR}\cos(\Omega\tau_{12}) e^{-\frac{\gamma_{\rm ph}-2S_{HR}\gamma_0}{2}\tau_{12}} \left(e^{-S_{HR}\gamma_0\tau_{12}} + 2 + e^{-\gamma_{\rm ph}\tau_{12}}\right) \\ & + S_{HR}\cos(2\Omega\tau_{12}) e^{-(\gamma_{\rm ph}-S_{HR}\gamma_0)\tau_{12}} \Bigg] \equiv \Psi_0(\tau_{12};\Omega) .\end{aligned} \tag{53}$$

In the main text, the Fourier transform of the photon echo signal is discussed. One may note that Eq. (53) is the sum of exponential terms of the form

$$E_1(t;\gamma_i) = e^{-\gamma_i t} , \quad E_2(t;\gamma_i,\Omega) = e^{-\gamma_i t}\cos(\Omega t) . \tag{54}$$

Straightforward calculation gives for their Fourier transforms:

$$\begin{aligned} F_1(\omega;\gamma_i) &= \int_0^\infty E_1(t;\gamma_i) e^{-i\omega t} {\rm d}t = \frac{1}{\gamma_i + i\omega} , \\ F_2(\omega;\gamma_i,\Omega) &= \frac{1/2}{\gamma_i + i(\omega - \Omega)} + \frac{1/2}{\gamma_i + i(\omega + \Omega)} \end{aligned} \tag{55}$$

As a result, the real part of the Fourier transform of echo signal is dominated by the sum of a few Lorentzians with different widths. In addition to the main narrow Lorentzian with the half width at half maximum (HWHM) of $(1 + S_H R)\gamma_0$ at zero frequency there are a few contributions with the amplitudes proportional to the Huang-Rhys factor. These contributions have larger broadening which is determined by $\gamma_{\rm ph}$ (for each phonon mode): (i) at zero frequency; (ii) at the phonon frequency Ω; and (iii) at the double phonon frequency 2Ω. Note that, under the theoretical assumptions, the amplitude of the peak at the double frequency is not determined by nonlinear processes

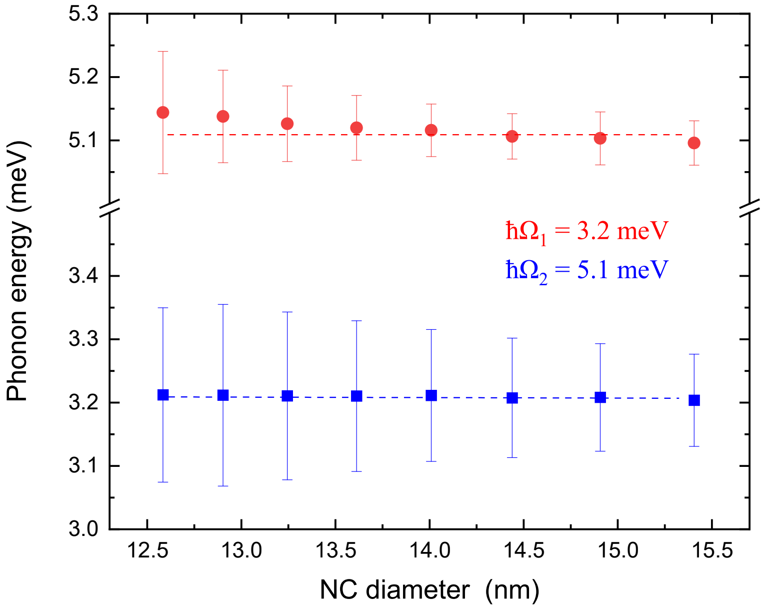

Supplementary Fig. 2 Depenedence of phonon energies $\hbar\Omega_i$ on the diameter of the NCs. The energies are obtained from the same fitting procedure of PE transients used to extract S_{HR} and τ_{ph} in Figure 4 in the main text. The error bars correspond to the width of the peaks given by $\pm\hbar/\tau_{\mathrm{ph}}$.

in the NCs, but by the intrinsic nonlinear nature of the photon echo signal. The amplitudes of the zero- and second-harmonic Lorentzians, with HWHM= $\gamma_{\mathrm{ph}} - S_{\mathrm{HR}}\gamma_0$ are approximately 4 times smaller than the amplitude of the peak at the fundamental phonon frequency.

Supplementary Note 6 Size dependence of phonon energies

The nanocrystals under study are relatively large, as a result the quantum confinement is small and the phonon modes have wave vectors near the Brillouin-zone center on the bulk phonon dispersion. Given the weak dependence of the energy of optical phonons near the Brillouin zone center, we expect a constant energy for the phonons discussed in this work in the whole range of NC sizes. This conclusion is supported by experimental data shown in Figure 2 which gives the dependence of the oscillation frequency (in energy units) as a function of the nanocrystal size. These energies are extracted from the same fitting of the time dynamics as the data in Figure 4 in the main text. As can be seen, the dependence of the oscillation frequency on the NC size is negligible within the the linewidths $\pm\hbar/\tau_{\mathrm{ph}}$ of the Fourier peaks which are shown by vertical bars.

Supplementary References

[1] Protesescu, L. *et al.* Nanocrystals of cesium lead halide perovskites ($CsPbX_3$, X = Cl, Br, and I): Novel optoelectronic materials showing bright emission with wide color gamut. *Nano Letters* **15**, 3692–3696 (2015).

[2] Kolobkova, E. V., Kuznetsova, M. S. & Nikonorov, N. V. Perovskite CsPbX3 (X=Cl, Br, I) Nanocrystals in fluorophosphate glasses. *Journal of Non-Crystalline Solids* **563**, 120811 (2021).

[3] Nestoklon, M. O. *et al.* Tailoring the electron and hole Landé factors in lead halide perovskite nanocrystals by quantum confinement and halide exchange. *Nano Letters* **23**, 8218–8224 (2023).

[4] Meliakov, S. R. *et al.* Landé g-factors of electrons and holes strongly confined in CsPbI3 perovskite nanocrystals in glass. *Nanoscale* **17**, 6522–6529 (2025).

[5] Harkort, C. *et al.* Confined acoustic phonons in $CsPbI_3$ nanocrystals explored by resonant Raman scattering on excitons. *Nano Lett.* **25**, 12754 (2025).

[6] Meliakov, S. R. *et al.* Hyperfine interaction of electrons confined in $cspbi_3$ nanocrystals with nuclear spin fluctuations. *Phys. Rev. B* **113**, 035304 (2026).

[7] Meliakov, S. R. *et al.* Temperature dependence of the electron and hole landé g-factors in $CsPbI_3$ nanocrystals embedded in a glass matrix. *Nanoscale* **16**, 21496–21505 (2024).

[8] Kirstein, E. *et al.* Mode locking of hole spin coherences in $CsPb(Cl,Br)_3$ perovskite nanocrystals. *Nature Communications* **14**, 699 (2023).

[9] Kuznetsova, M. S. *et al.* Synthesis and optical properties of perovskite nanocrystals in glass with cationic substitution. *Journal of Chemical Physics* **161**, 124501 (2024).

[10] Utzat, H. *et al.* Coherent single-photon emission from colloidal lead halide perovskite quantum dots. *Science* **363**, 1068–1072 (2019).

[11] Lv, Y. *et al.* Exciton-acoustic phonon coupling revealed by resonant excitation of single perovskite nanocrystals. *Nature Communications* **12**, 2192 (2021).

[12] Tamarat, P. *et al.* The dark exciton ground state promotes photon-pair emission in individual perovskite nanocrystals. *Nature Communications* **11**, 6001 (2020).

[13] Becker, M. A. *et al.* Long exciton dephasing time and coherent phonon coupling in $CsPbBr_2Cl$ perovskite nanocrystals. *Nano Letters* **18**, 7546–7551 (2018).

[14] Liu, A. *et al.* Multidimensional coherent spectroscopy reveals triplet state coherences in cesium lead-halide perovskite nanocrystals. *Science Advances* **7**,

eabb3594 (2021).

[15] Han, Y. *et al.* Lattice distortion inducing exciton splitting and coherent quantum beating in CsPbI3 perovskite quantum dots. *Nature Materials* **21**, 1282–1289 (2022).

[16] Varshalovich, D. A., Moskalev, A. N. & Khersonskii, V. K. *Quantum theory of angular momentum* (World Scientific, 1988).

[17] Miyata, K. *et al.* Large polarons in lead halide perovskites. *Science Advances* **3**, e1701217 (2017).

[18] Thouin, F. *et al.* Phonon coherences reveal the polaronic character of excitons in two-dimensional lead halide perovskites. *Nature Materials* **18**, 349–356 (2019).

[19] Rainò, G. *et al.* Superfluorescence from lead halide perovskite quantum dot superlattices. *Nature* **563**, 671–675 (2018).

[20] Qian, C. *et al.* Nanoscale phonon dynamics in self-assembled nanoparticle lattices. *Nature Materials* **24**, 1616–1625 (2025).

[21] Matsuura, M. & Büttner, H. Optical properties of excitons in polar semiconductors: Energies, oscillator strengths, and phonon side bands. *Phys. Rev. B* **21**, 679–691 (1980).

[22] Iadonisi, G. & Bassani, F. Excitonic polaron states and optical transitions. *Il Nuovo Cimento D* **2**, 1541–1560 (1983).

[23] Berman, P. R. & Malinovsky, V. S. *Principles of Laser Spectroscopy and Quantum Optics* (Princeton University Press, 2011).

[24] Poltavtsev, S. V. *et al.* Polarimetry of photon echo on charged and neutral excitons in semiconductor quantum wells. *Scientific Reports* **9**, 5666 (2019).
